# Supplementary material for: Optomechanical mass spectrometry
Source: Nat Commun. 2020 Jul 29;11:3781. doi: 10.1038/s41467-020-17592-9 (PMC7391691; doi:10.1038/s41467-020-17592-9)
Supplement: Supplementary file 1 — Supplementary Information [file 41467_2020_17592_MOESM1_ESM.pdf]

Supplementary Information for:

Optomechanical mass spectrometry

Sansa and Defoort et al.

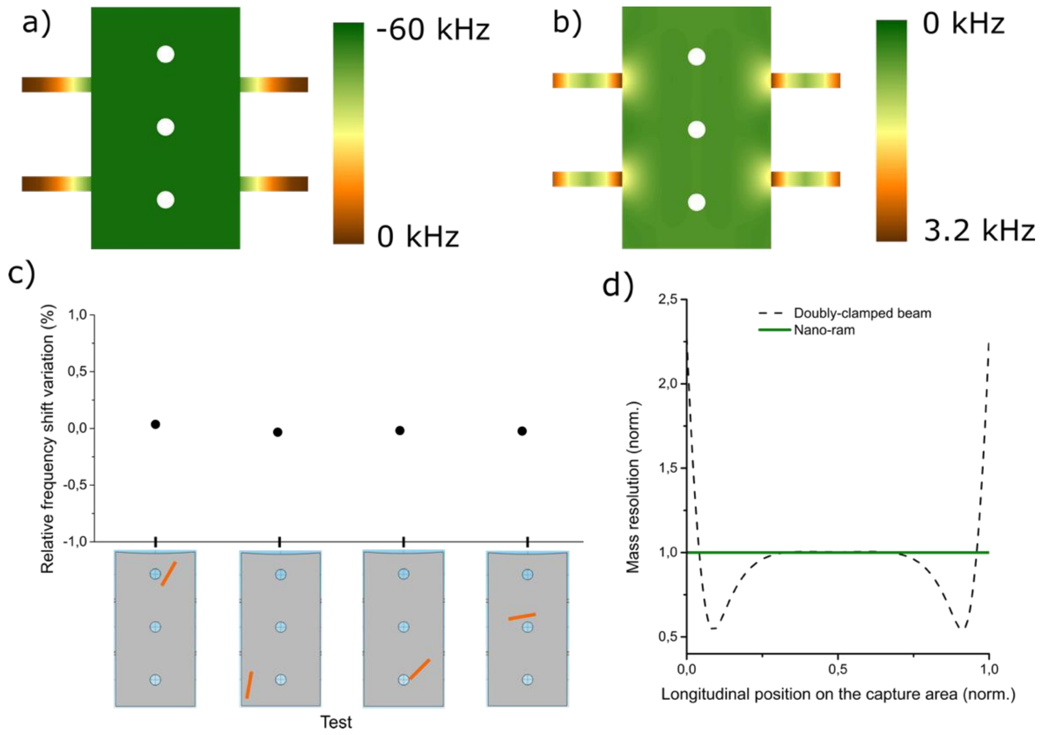

Supplementary Figure 1. Finite Element simulation of nanoresonator frequency shifts induced by deposition of particles with various properties. a) Frequency shift caused by deposition of tantalum nano-sphere of 25 nm radius at different positions on the nanoresonator. This shift is insensitive to particle position on the sensing platform within numerical error. b) Difference in frequency shift induced by a two orders of magnitude change in particle stiffness, from 1 GPa to 100 GPa. c) Frequency shift induced by deposition of a nano-rod (orange, 25 nm radius, 500 nm long) at four different positions on the platform. This accounts for less than 0.05 % of the frequency shift, which is orders of magnitude below our current resolution. (d) Mass resolution of a doubly clamped beam showing the large dependency on the landing position due to the two-mode operation (black, dashed line). With single-mode operation, the nano-ram features a flat mass resolution over the whole sensing platform (green, full line). Note that the four clamped supports of the nanoresonator are very sensitive to landing position and stiffness, it is therefore necessary to make their capture area negligible compared to that of the platform, or to protect them from any deposition as it is performed in this work.

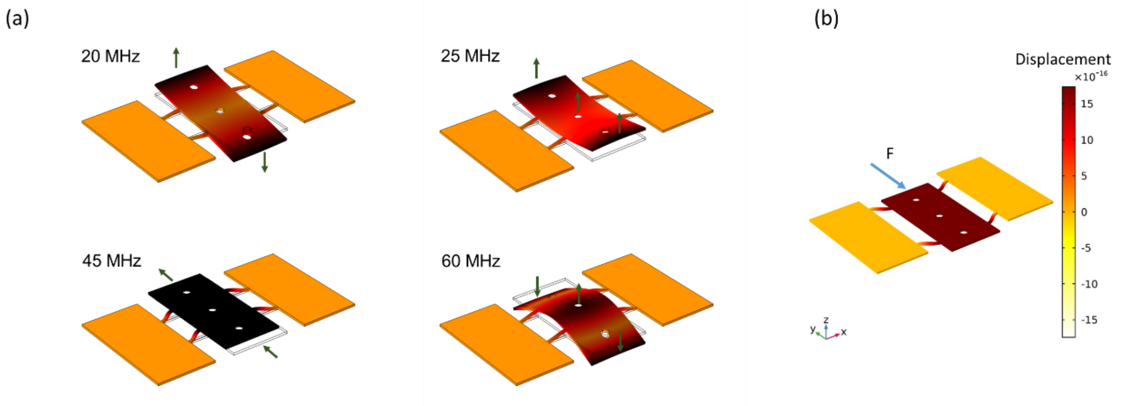

Supplementary Figure 2. Finite element modal simulations of the nanomechanical resonator, using Comsol Multiphysics. (a) Modal simulation of the structure. The orange parts are clamped. The green arrows indicate the general motion of the resonator as a guide to the eye. The simulated modes are well separated in frequency. The in-plane mode of interest for mass sensing is around 45 MHz. Note that the vibration mode at 60 MHz presents nodes of vibration close to the clamps, while the mode at 25 MHz follows a semi-rigid out-of-plane motion. (b) Static simulations to extract stiffness and effective mass of the structure by applying a constant force and solving for displacement. The obtained stiffness is 48.5 N/m. Combined with the resonance frequency extracted from modal simulations, we obtain an effective mass of 5.8×10^{-16} kg, which agrees to 99.3% with the mass of the platform as determined from its dimensions and density.

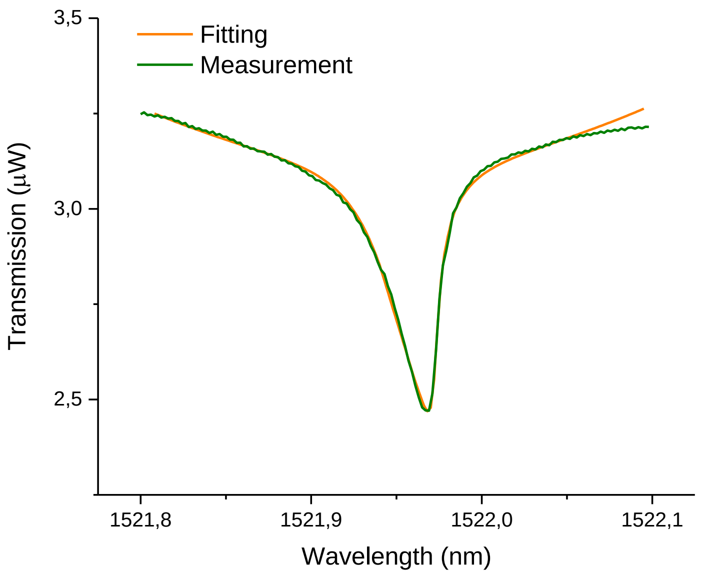

Supplementary Figure 3. Fitting of the optical response of one mode of the optical resonator. The laser output power is 0.5 μW, and the optical response shows a slight thermo-optic effect, resulting in an asymmetric mode shape. We fit the measurement data using a model which takes into account this effect[1]. We obtain an intrinsic quality factor of $Q_\mathrm{i} = 5 \times 10^4$ and an extrinsic quality factor $Q_\mathrm{e} = 8 \times 10^5$ (under-coupled regime).

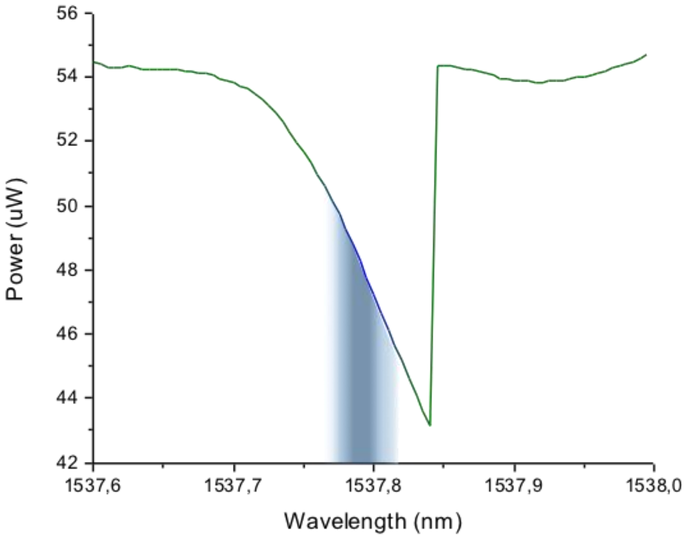

Supplementary Figure 4. Narrow-band optical transmission spectrum of the ring resonator. Detail of one resonance of the ring resonator measured with 10 mW output laser power, in order to maximize optomechanical signal amplitude. A strong thermo-optic behavior is observed, shifting the resonance to higher wavelengths. The wavelength of operation is chosen within the linear slope in the blue area so the transmission signal is maximized. The wavelength modulation induced by the motion of the nanoresonator remains small compared to the width of the optical response (typically 2 pm), even for large mechanical drive amplitudes (Figure 2c).

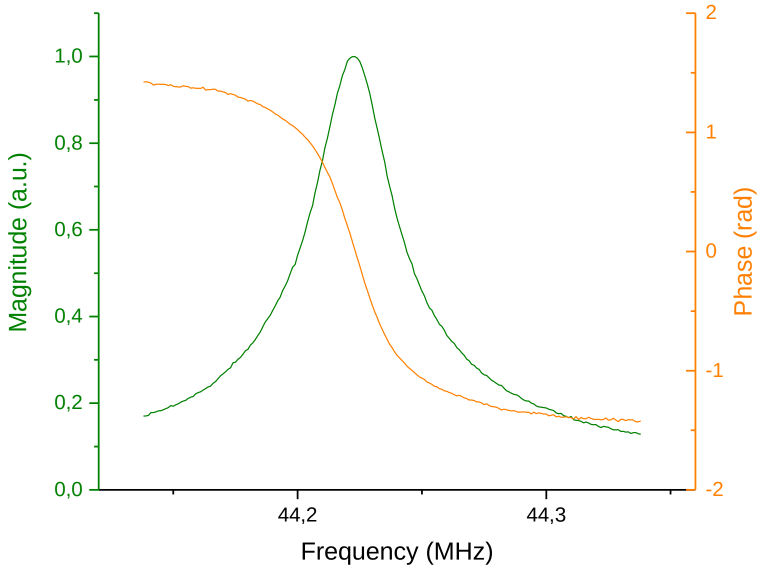

Supplementary Figure 5. Response of the nanomechanical resonator in magnitude and phase. The measurements are performed at approximately -3 dB from the onset on non-linearity. The phase response shows a close to π rad shift, as expected from a pure harmonic oscillator with no parasitic signals. This is the result of good decoupling between electrical actuation and optical detection.

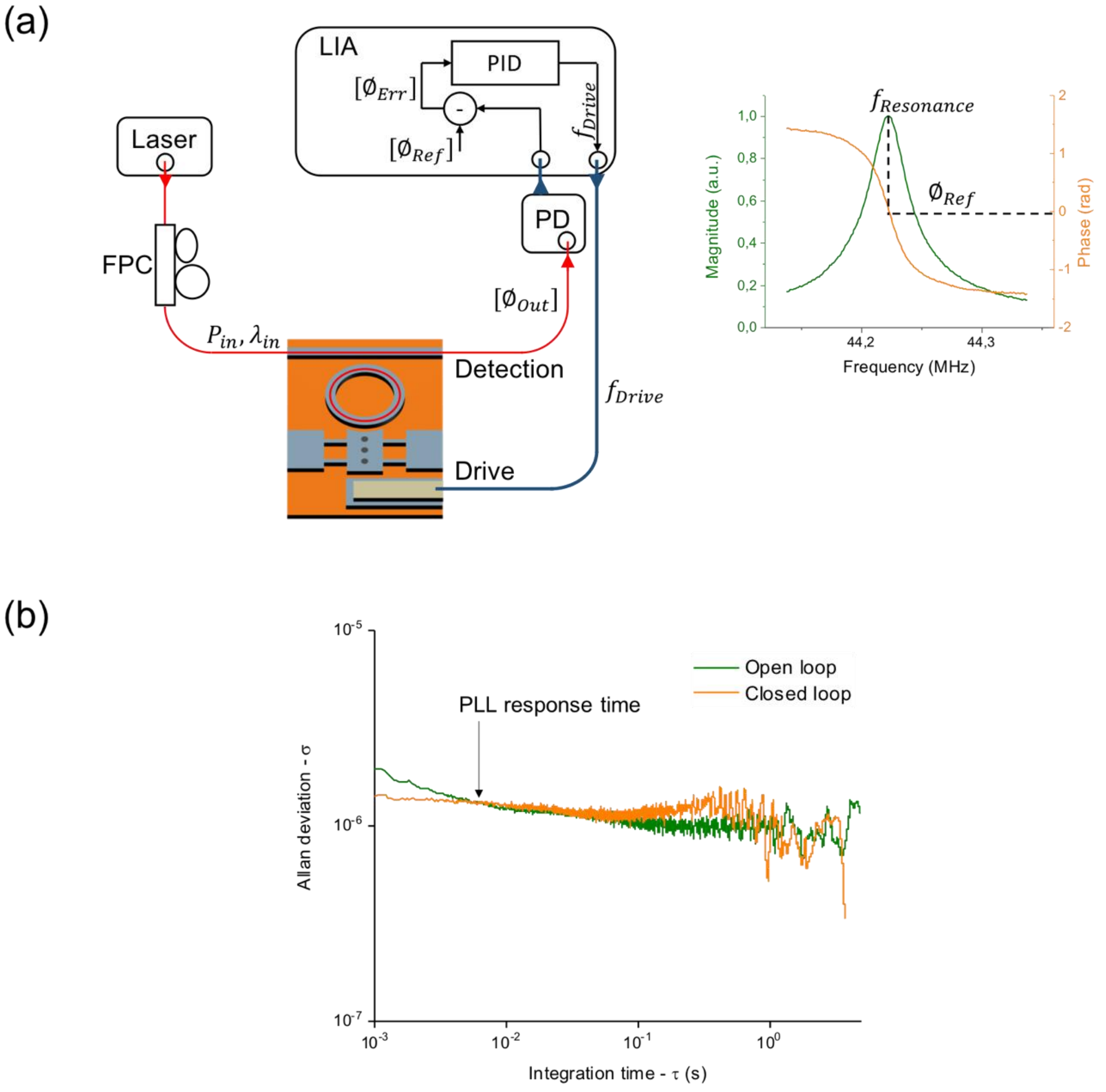

Supplementary Figure 6. Frequency stability measurement. (a) Schematic of closed loop measurements. A PID is used to lock the driving frequency to the phase of the resonator, in a phase locked loop (PLL) configuration. The drive frequency is changed so that $f_{\text{Drive}} = f_{\text{Resonance}}$. This is done by reading the output phase of the resonator and comparing it to its phase at resonance $\emptyset_{\text{Ref}}$ to obtain a phase error $\emptyset_{\text{Err}}$. The PID modifies the driving frequency to minimize this phase error. In our case, the PLL is embedded in our lock-in. (b) Allan deviation of the nanomechanical resonator in open loop and closed loop configurations. The open loop measurements are performed by driving the resonator at resonance and monitoring its phase fluctuations, which are later converted to resonance frequency fluctuations using the linear phase-to-frequency relationship of the resonator close to resonance $\Delta\phi/\Delta f \cong 2Q/f_0$. This frequency trace is then used to compute the Allan deviation. In the case of closed loop measurements, the Allan deviation is directly computed from the drive frequency. Both measurements provide very similar results down to an integration time of 10^{-2} seconds, which is the response time of our PLL, confirming correct frequency tracking in closed-loop operation. At lower integration times, the frequency is filtered by the PI corrector and the device's resonance frequency is not tracked correctly. At large integration times (typically a few seconds), a drift in the resonance frequency becomes visible, which we attribute to temperature fluctuations. As discussed in Methods, the integration time is chosen close to the PLL response time (~10ms) in order to avoid multiple event detection.

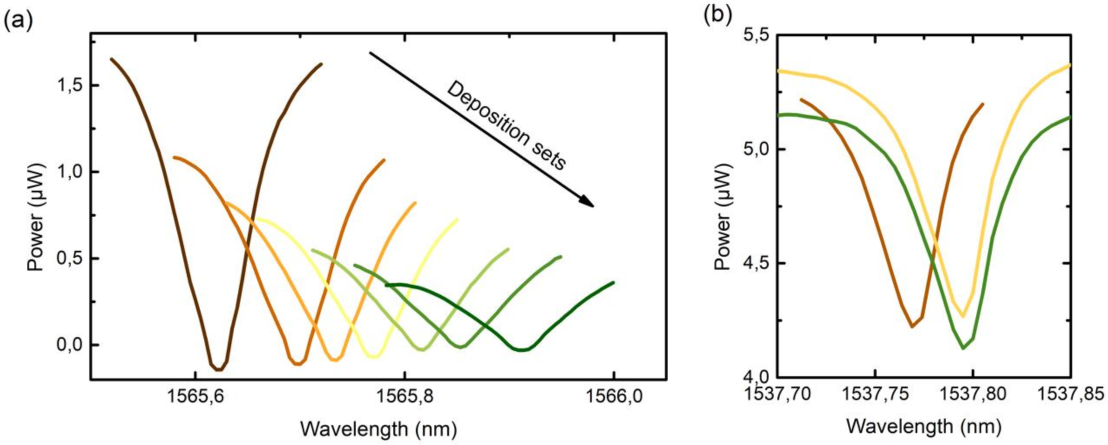

Supplementary Figure 7. Effect of mass deposition on the optical transmission with and without protection layer. (a) Without protective layer, as tantalum particles are deposited on the waveguide and the ring, their optical properties drift. The resonance shifts towards higher wavelengths and the optical transmission of the waveguide degrades. In particular, the slope decreases, degrading the optomechanical transduction gain. (b) Conversely, when the optical part is covered with an amorphous silicon layer, no clear degradation of the optical readout due to particle deposition is observed. Even after depositing the equivalent of 10% of the nanoresonator's mass (brown: before any deposition, orange: between depositions, green; after depositions), the optical resonance wavelength remained within its bandwidth with no discernible change in the transmission.

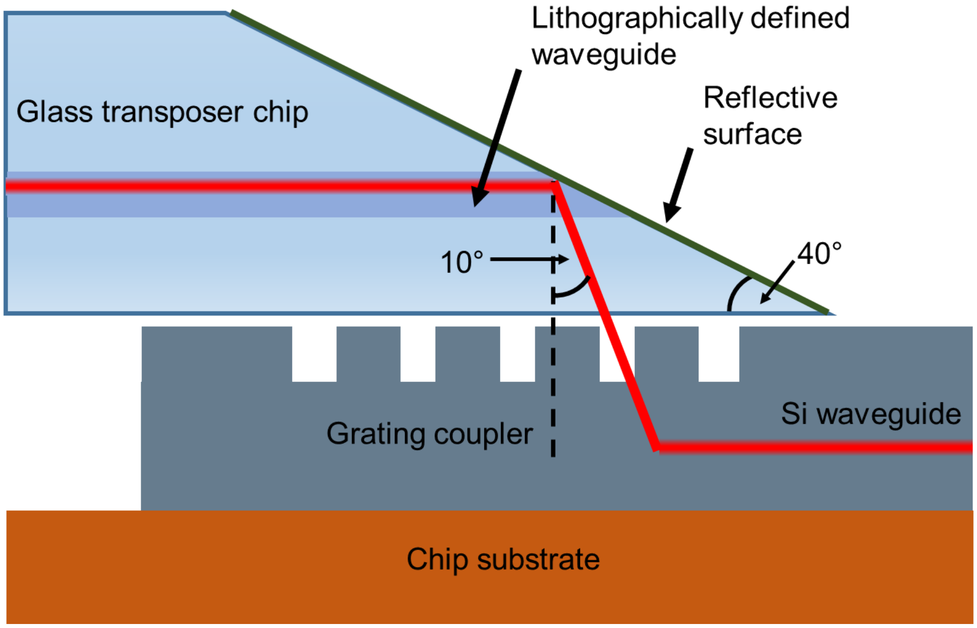

Supplementary Figure 8. Schematic of the optical packaging using fiber-to-waveguide transposer chips, fabricated by Teem Photonics. The transposer chips are fabricated from glass, where optical waveguides are realized using photolithography and ion implantation[2]. This fabrication process results in narrow waveguides, which can be placed very close together in the chip: this allows a large number of densely packed interconnections. Moreover, as opposed to more standard top-coupling techniques such as butt-coupling[3], this provides quasi-planar connections with small footprint. The light is coupled into the transposer chips on one end from standard FC/APC mono-mode optical fibers and travels through the implanted waveguides, which are terminated by a reflective surface at a certain angle. The light is reflected towards the silicon grating couplers. The ion-defined waveguides in the transposer chips are less than 10 μm away from the bottom surface, therefore minimizing the light beam divergence and resulting in high efficiency coupling. The grating couplers are designed to have an optimum input angle of 10 degrees, so the angle of the reflective surface is chosen to be 40° during the fabrication of the chips. The size of the transposer chips is approximately 1 mm × 2 mm × 20 mm. Both transposer chips are aligned to maximize light transmission, and then glued to the optomechanical chip.

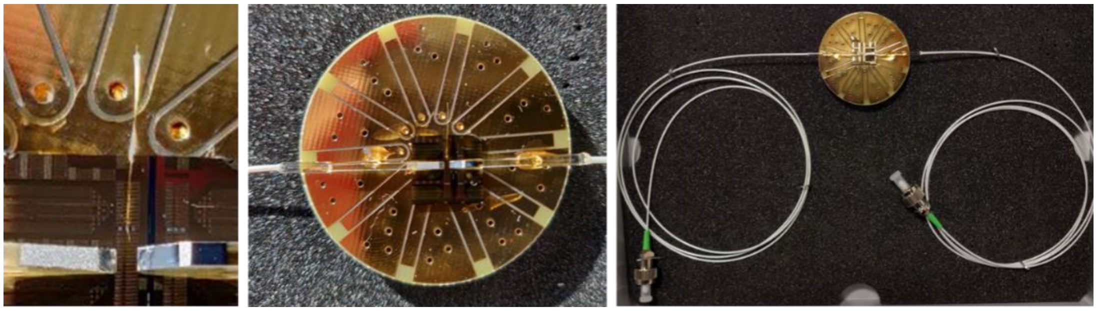

Supplementary Figure 9. Packaging of the optomechanical devices with electrical actuation. Left: detail showing the optomechanical chip with the nanoresonators, the two transposer chips and the electrical wire bonding to the PCB; middle: PCB with the optomechanical chip; right: general view showing the whole assembly, with standard single mode optical fibers with FC/APC terminations. The whole packaging process is as follows: first, the optomechanical devices are assembled with the fibre-to-waveguide transposer chips. Then the assembly is glued to a PCB, and the wire-bonding of the electrical connections is performed. The PCB has electrical pins at the bottom (not shown in the image), which are plugged to the measurement system. In this example, the two transposer chip extremities are less than 1 mm away from each, and less than 500 µm from the optomechanical resonator.

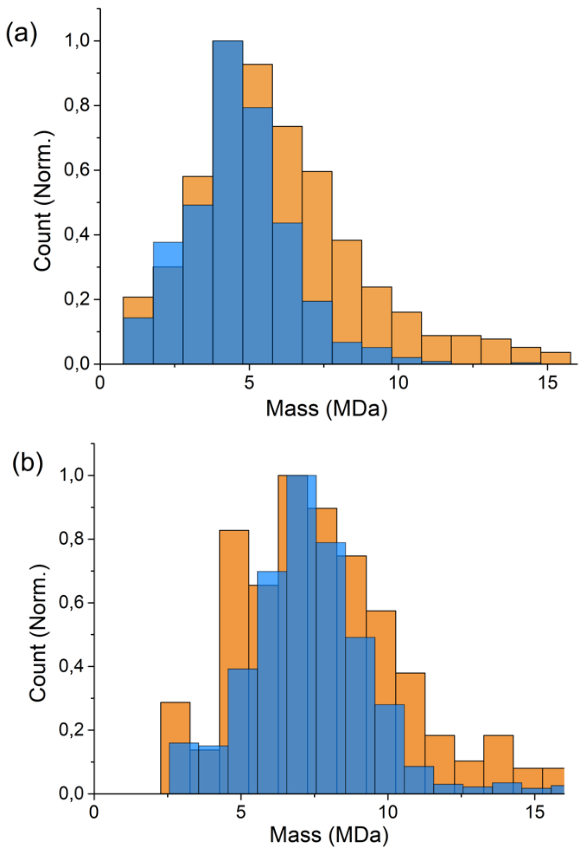

Supplementary Figure 10. Measurement compared to simulation. Experimental (orange) compared to theoretical (blue) mass measurements for (a) 4.6 MDa and (b) 6.8 MDa particles. The blue spectra are obtained by simulating deposition of monodisperse populations on a nanoresonator using the measured frequency noise fluctuations. The same data processing has been used for the four data sets of the main text. The non-zero width of the blue spectra are due to the nanoresonator's frequency noise. The experimental width being larger than the theoretical one, the measurements reflect the actual distribution of the tantalum population.

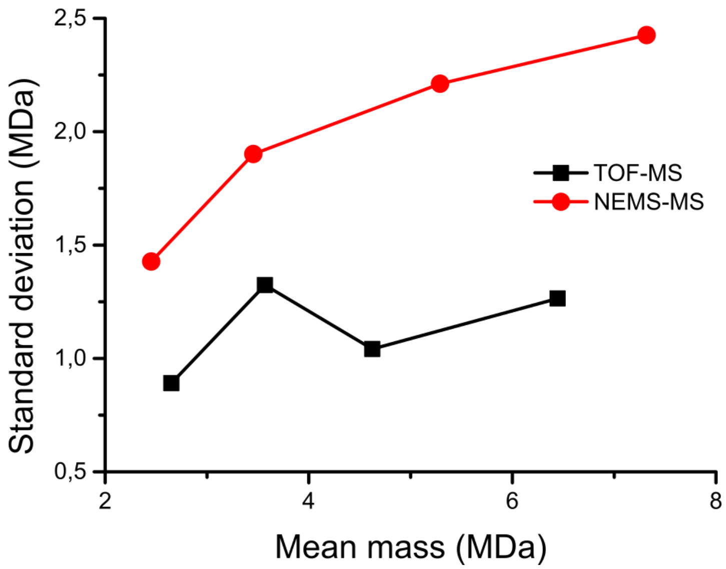

Supplementary Figure 11. Mass distribution width (expressed in standard deviation) vs increasing mean mass. The results obtained with optomechanical mass spectrometry captures a clear trend when population mass increases and shows the population dispersion increases. Conversely, TOF-MS efficiency at high masses dramatically drops and the TOF fails to account for this width increase at high masses.

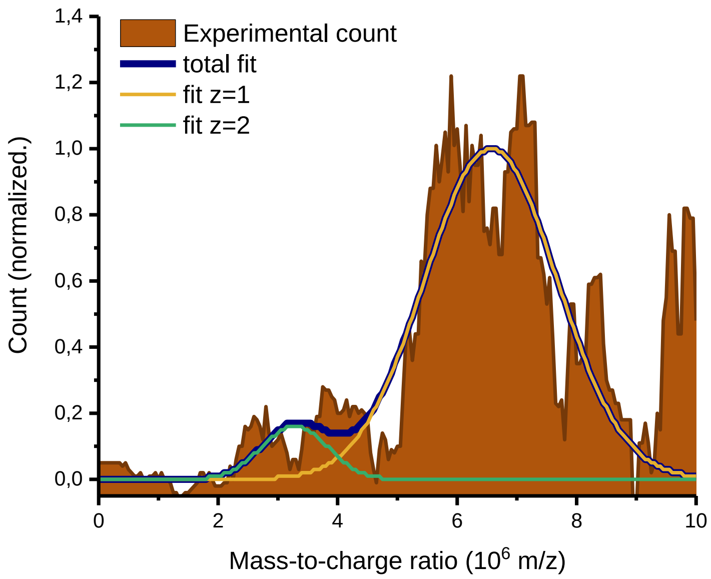

Supplementary Figure 12. Highest mass TOF-MS spectrum (6.57 MDa in Fig. 4b) over a large mass span for a better view: different charge states can be fitted in the spectrum (here, one and two charges).

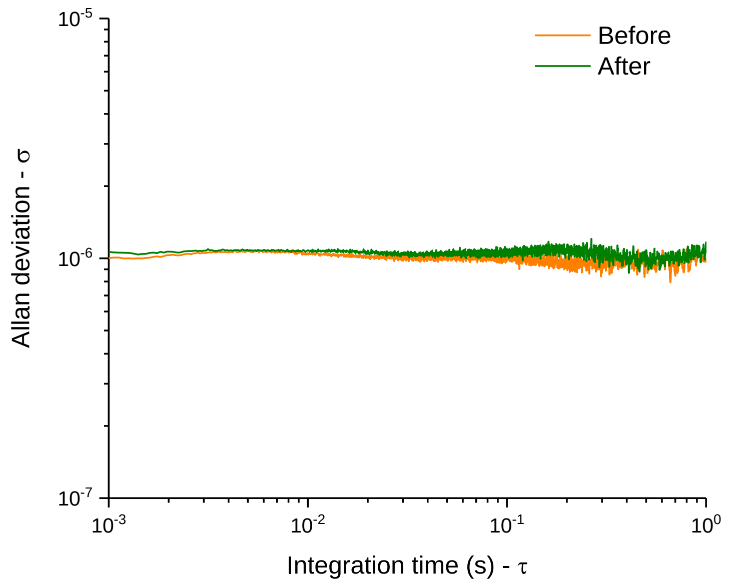

Supplementary Figure 13. Measurement of the closed-loop frequency stability before and after a mass deposition run. No degradation is observed after 300 s deposition of 5.7 MDa particles. This is representative of all our mass measurements; no degradation of the frequency stability is observed after deposition of up to 10 % of the mass of the nanoresonator.

Supplementary note 1: optical characterization

Optical resonators. The optical resonators are silicon rings supported by four spokes connected to a central disk itself clamped to the substrate by a SiO_2 pillar (Fig. 1c of the main text). The thickness of the ring resonators and optical waveguides is 220 nm, in order to limit optical losses in silicon. The rings have a 20 μm external diameter and a 0.5 μm width. The four spokes are 500 nm wide except at their extremity in contact with the ring where they are ~100 nm wide in order to decrease optical losses.

Estimation of the optomechanical coupling. The optomechanical coupling factor g_{om} is estimated using the thermomechanical noise of the nanoresonator as calibration [4]. We define the optical transmission spectrum of the optical resonator as measured by the photodetector by a transfer function $T(f)$ [V] which depends on the frequency of the laser light f. When the ring is coupled to a nanoresonator vibrating with an amplitude $x_{\mathrm{NR,m}}$ $[m]$, it will shift the optical frequency of the cavity by a certain amount defined by the optomechanical coupling factor g_{om} []. When light with frequency f_0 is coupled to the optical cavity, the nanoresonator's motion modulates the photodetector output voltage as follows: $x_{\mathrm{NR,V}} = x_{\mathrm{NR,m}}\, g_{\mathrm{om}} \left.\frac{\delta T(f)}{\delta f}\right|_{f_0}$ [V] (see inset of Fig. 2a of the main text for a schematic representation of this effect). Therefore, for a given measurement of the nanoresonator's motion, one can obtain the optomechanical coupling factor if the corresponding motion of the nanoresonator (in m) is known, as well as the slope of the optical response (which is easily measured).

We used the thermomechanical noise of the nanoresonator, which provides a known $X_{\mathrm{NR,m}}$ $[m\, Hz^{-1/2}]$. We performed a thermomechanical noise measurement (inset of the Fig. 2c of the main text), which provides $X_{\mathrm{NR,V}}$ $[V\, Hz^{-1/2}]$. The theoretical thermomechanical noise is equal to $X_{\mathrm{NR,m}} = \sqrt{\frac{4\mathrm{k_B TQ}}{2\pi \mathrm{m_{eff}} \omega_0^3}}$ $[m\, Hz^{-1/2}]$, in our case $1.03 \times 10^{14}\ m\, Hz^{-1/2}$. We then measured the slope of the optical response $\frac{\delta T(f)}{\delta f}$ in the same measurement conditions, which was $14.9\, kV\, m^{-1}$. For these calculations, we assumed the optical response to be linear around the light frequency f_0, and that the slope measured during a slow spectrum characterization is representative of that during the nanoresonator's measurements at high frequency. From these measurements we determined $g_{om} = 4.05 \times 10^{17} Hz\, m^{-1}$ $(0.405\ GHz\, nm^{-1})$.

Supplementary References

1. Schwab, L. *et al.* Comprehensive optical losses investigation of VLSI Silicon optomechanical ring resonator sensors. in *2018 IEEE International Electron Devices Meeting (IEDM)* 4.7.1-4.7.4 (2018).
2. Magazine, P. I. C. PICs: a little piece of glass can change everything - PIC Magazine News. *PIC* /article/101217/PICs_A_Little_Piece_Of_Glass_Can_Change_Everything/feature.
3. Bernabé, S. *et al.* A fully packaged 25 Gbps/channel WDM photoreceiver module based on a Silicon Photonic Integrated Circuit and a flip-chipped CMOS quad transimpedance amplifier. in *2016 IEEE Optical Interconnects Conference (OI)* 4–5 (2016).
4. Gorodetksy, M. L., Schliesser, A., Anetsberger, G., Deleglise, S. & Kippenberg, T. J. Determination of the vacuum optomechanical coupling rate using frequency noise calibration. *Opt. Express, OE* **18**, 23236–23246 (2010).
